# Effect of an additional floating electrode on radio frequency cross-field atmospheric pressure plasma jet

**DOI:** 10.1038/s41598-023-37805-7

**Published:** 2023-07-01

**Authors:** Radhika T. P., Satyananda Kar

**Affiliations:** Department of Energy Science and Engineering, Indian Institute of Technology Delhi, Hauz Khas, New Delhi, 110016 India

**Keywords:** Applied physics, Plasma physics

## Abstract

Atmospheric pressure plasma jets with cross-field electrode configuration are a potential jet design for gases with high breakdown fields. This study focuses on the effect of an additional floating electrode on the cross-field plasma jet properties. Detailed experiments are done with the additional floating electrodes of different widths introduced below the ground electrode in a plasma jet with a cross-field electrode configuration. It is observed that in the presence of an additional floating electrode in the jet propagation path, less applied power is needed for the plasma jet to cross the nozzle and jet length increases. This threshold power, as well as the maximum jet length, depends on the electrode widths. A detailed analysis of charge dynamics in the presence of an additional floating electrode shows decrement in the net charge transferred radially to the external circuit through the ground electrode, and an increment in the net charge transferred axially. Increment in the optical emission intensity of reactive oxygen and nitrogen species, as well as the relative yield of ions like N$^{+}$, O$^{+}$, OH$^{+}$, NO$^{+}$, O$^{-}$, and OH$^{-}$ in the plasma plume, that are crucial for biomedical applications suggest an improvement in the reactivity of plasma plume in the presence of additional floating electrode.

## Introduction

Cold atmospheric pressure plasmas (CAP) has been an active area of research for more than 50 years as they do not require extensive vacuum systems for plasma generation and magnetic field for confinement and are proposed as cost-effective to existing low-pressure plasma processes^1^. These non-equilibrium plasmas have widely different electron and ion temperatures ($T_{e} \sim$ 6000 K, $T_{i} \sim 300$ K), offer high electron densities ($\sim 10^{16}$–$10^{19}$ m$^{-3}$ ) compared to conventional low-pressure plasmas^2^ and are an ideal source for a wide range of applications^3–5^. There are different types of devices for non-thermal plasma production such as atmospheric glow discharges, corona discharges, gliding arc discharges, dielectric barrier discharges (DBD), and atmospheric pressure plasma jets. The corona discharge produces near a high electric field around a sharp pointed metallic electrode. DBDs with dielectric or highly resistive material between two metal electrodes ignite a spatially constrained discharge with restricted free flow of high current between the electrodes. Atmospheric pressure plasma jets produce plasma that flows out of the discharge region into the ambient air. All these three are the most reliable sources for producing non-thermal plasma for different applications. DBDs are ideal for the homogeneous treatment of substrates as they generate a large number of micro discharges between electrode gap^6^ and are widely used for flow control^7^ and aerodynamic drag reduction^8^. Atmospheric Pressure Plasma jets (APPJ) have attracted recent research interest among the sources of non-equilibrium cold plasmas as they offer the practical capability to perform as a plasma chemical reactor where reactions occur inside the discharge unit as well as in the plasma jet surrounding air. Interaction of the charged particles in this plasma with the ambient air produces reactive oxygen and nitrogen species (RONS)^9,10^, and their low gas temperature provides a conductive environment for the interaction with the biological cells and tissues^11^. All these peculiarities of APPJ suggest them as a potential candidate for biomedical applications^12–15^, surface treatment and modification^16,17^, thin film deposition^18,19^, food technology^20,21^, and environmental applications^22^.

Plasma jets ignited by radio frequency are proven to be more efficient in terms of power coupling and require relatively lower breakdown voltage compared to other sources such as pulsed DC and AC^23^. Being the lighter element, electrons respond instantaneously to RF fields and convert the oscillatory energy gained from the RF field to random energy through collisions with neutral gas atoms. At the MHz frequency range, the alternating field may reverse some or all the electrons before they lose to the anode. This will increase ionization as the electrons move back and forth in the oscillating RF field and will make up the electron lost to the wall and electrodes^24,25^. The high concentration of highly energetic electrons in RF plasma compared to low-frequency plasmas enhance the production of reactive oxygen and nitrogen species which is necessary for biomedical and material processing applications^23^. The high electric field in the inter-electrode region suggests cross-field jets as an effective jet design for gases with high breakdown voltages. When the highly oscillating RF field is applied to a jet with the cross-field electrode configuration, a radially directed momentum will impart on the electrons which will restrict the axial movement of electrons^26^. This spatially confined ionization offers a shorter and less reactive plasma plume for RF cross-field jets compared to AC cross-field plasma jets^27^. This study aims to improve this short and less reactive plume of radio-frequency cross-field plasma jet by introducing an additional floating electrode.

The two important factors of interest for all kinds of applications are the interaction rate of the plasma plume with the ambient air as well as with the target and RONS concentration. For better results and efficacy, a sufficient plasma jet length in the ambient air and good chemical reactivity of the plasma plume are desired. Plasma jet properties can control with external factors like gas flow rate^28,29^, gas mixing ratio^30^, and frequency, amplitude, and waveform of the applied voltage^31–34^. Electrode configuration is another factor that has a prominent influence on plasma parameters^35,36^. It is reported that multi-electrode jet systems with alternate power and ground electrodes form plasma jets with enhanced OH density in the inter-electrode space, though the jet length and emission intensity are reduced^37^. An additional ground electrode to a two-electrode linear-field system enhances the emission intensity, and the laminar to turbulent transition occurs at a higher flow rate^38^. Adding a second ground electrode to a linear field system causes a higher accumulation of charges on the inner wall of the quartz tube of the plasma jet and increases the discharge current and dissipated power^39^. Multi-electrode DBD plasma actuators are studied with an aim to create stronger discharge which will lead to higher resultant force^40^. Electrode position is also an important factor for APPJs. An increase in distance between the ground and high-voltage electrode results in changes in discharge mode and discharge parameters^41^. For plasma jets with power-floating electrode configurations, RONS are abundantly generated in the downstream region and near the treating target, resulting in a better-treating efficiency than the jets with power-ground electrode configuration where active species are trapped between electrodes^42^. Additional floating rings in the propagation path of the linear-field jet result in a shorter plasma jet as the electric field is perturbed (decreased) by the charges accumulated on the dielectric wall^43^. An additional floating pin electrode covered by a glass tube and a powered ring electrode outside the glass tube increases the electric field and decreases the voltage needed to trigger the plasma jet^44^. Extensive research has been going on the effect of electrode configurations on plasma properties.

This study focuses on the effect of an additional floating electrode in the plasma jet propagation path to improve the short and less reactive plasma plume of a radio frequency (RF) induced cross-field jet configuration. The experiments have been done for the net charge moving axially, net charge transferred radially from the electrode gap to the external circuit, discharge power, and floating potential fluctuations in the plasma plume due to charge redistribution in the presence of an additional floating electrode to improve the plasma jet length and reactivity.

## Experimental set up

**Figure 1:**
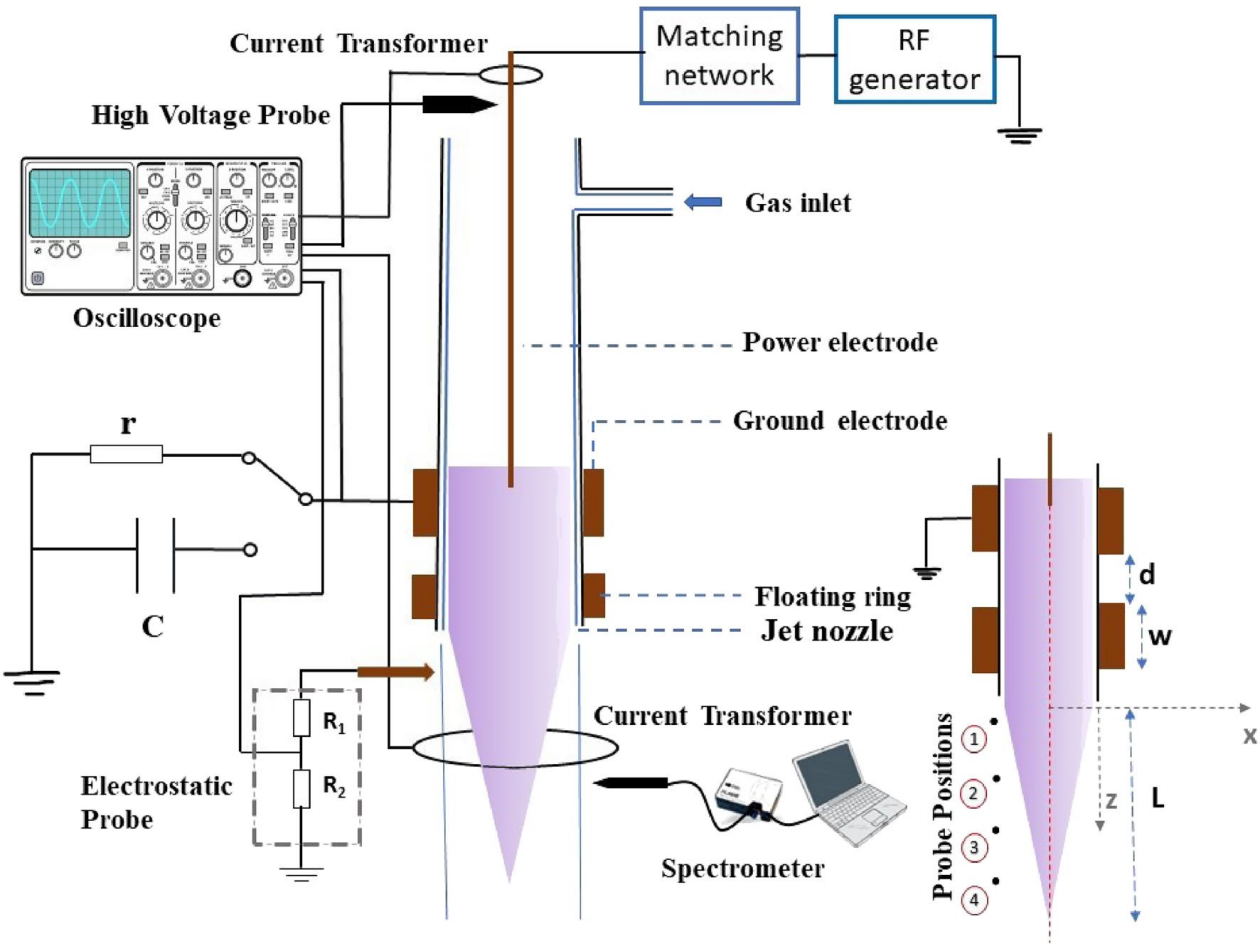
Schematics of the atmospheric pressure plasma jet design.

Figure 1 shows the schematics of the experimental setup. The plasma jet was created inside a Pyrex glass tube employing a pin-to-ring electrode arrangement. The glass tube has an inner diameter of 6 mm with a 2 mm thickness wall. A copper power electrode with a diameter of 1.6 mm was introduced at the centre of the glass tube. A copper strip of width 3 mm was used as the ground electrode and wrapped around the glass tube. Argon gas was the discharge gas with a flow rate of 3 lpm. The jet has a cross-field electrode configuration since the electric and gas flow fields are perpendicular to each other. A 13.56 MHz radio frequency (RF) power supply is connected to the plasma reactor through a matching network.The matching network (AIT-600) has a standard L-type topology and will transfer the load impedance to 50 $\Omega$ to ensure maximum power transfer to the load^45^. RF power supplied to the power electrode ignited the discharge and flowed as a plasma jet to the ambient air. Concentric floating copper rings of width *w* were placed between the ground electrode and the tube nozzle at a fixed distance *d* from the ground electrode aiming to improve the plasma jet length and reactivity.

A high voltage probe (Tektronix P6015A) and a current transformer (Pearson Electronics 8590C) were connected to the power electrode to measure the input voltage and current, respectively and the actual power being fed to the plasma is calculated from these current and voltage waveforms scoped with a digital storage oscilloscope (KEYSIGHT DSOX3024T). A glass tube with the same diameter as the plasma reactor was introduced after the jet nozzle to avoid the chances of arcing and for safe assembly of the current transformer. The current transformer was then placed around this glass tube at a fixed distance from the nozzle to measure the plasma jet current. The net charge being transferred radially to the external circuit was measured across a series resistor to the ground electrode. The plasma discharge power was measured across a series capacitor connected to the ground electrode.

The concentration of RONS and the electromagnetic waves interacting with the target are two of the key parameters in biomedical applications of APPJ. For both the parameters electric field is a crucial factor^46,47^. The additional floating electrode may cause a redistribution of charges and will cause fluctuations in plasma potential and non-uniformity of the electric field. These have an intense effect on the transport and heating of active species, the adhesive ability of the surface, and the properties and shape of the plasma sheath on the plasma–liquid interface. A single-pin probe was used to measure the fluctuations in the floating potential caused by the charges of guided ionization waves^48–50^. The probe was made of a 50 $\Omega$ coaxial cable and the protruding internal conductor of the cable with a 1 mm diameter and length of 10 mm was used as the probe tip. The probe can measure the potential fluctuations in the radial and axial directions of the jet with a help of an XYZ linear stage. The probe was placed perpendicularly at 4 mm radially away from the central line of the jet and recorded the signal at four axial positions (z = 2, 8, 14, and 20 mm ) with an oscilloscope connected to the probe after a potential divider designed with suitable resistors.

The local emissivity of the plasma jet in the wavelength range of 200–900 nm was investigated with an optical emission spectrometer of resolution 0.9 nm (Ocean Optics HR4000) coupled with 200 $\upmu$m optical fiber cable. Optical emission spectroscopy (OES) gives a qualitative analysis of species composition in the plasma plume. A quantitative analysis of the same is done with Molecular Beam Mass Spectroscopy (MBMS) (HPR60 MBMS Hiden Analytics Ltd.). The plasma jet was aligned perpendicular to the MBMS orifice at a fixed distance from the orifice (Supplementary Fig. 4) and recorded the data in the range of 1–100 amu (m/z). To reduce the noise induced by temporal variations of the plasma, the relative yield percentage of ions was used for the analysis^51^ of positive and negative ion species density.

## Experimental results and discussion

With this cross-field plasma jet configuration, plasma got ignited between two electrodes when the applied voltage is above the breakdown voltage and the jet flows out to the ambient air with higher applied power. The gas flow rate is optimized to 3 lpm for maximum jet length. There is a difference between the power measured at the source and the actual power being delivered to the load due to the several elements in the electrical equivalent of the jet assembly. The instantaneous power feeding to the load is monitored with the calibrated high-voltage probe and current transformer connected to the power electrode since the power delivered to the load is crucial in the electrical characterization of the plasma jet^52^. It is observed that for input powers of 85, 75, 65, and 55 W from the RF power supply display, the actual power being delivered to the load is $40.3\pm 0.3, 33.1\pm 0.3, 25.8\pm 0.4$, and $20.9\pm 0.3$ W, respectively.

**Figure 2:**
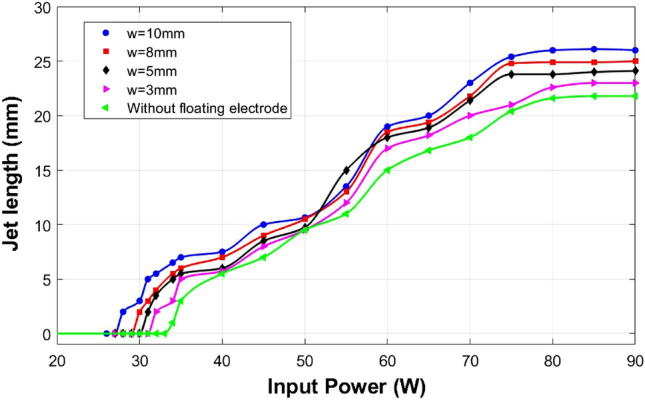
The plot of plasma jet length versus the applied power shows the variation in the jet length in the presence of floating electrodes of different widths at different applied powers.

Additional floating electrodes of different widths made of the copper strip are introduced below the ground electrode expecting an improvement in plasma jet length and reactivity. In the presence of an additional floating electrode on the plasma jet propagation path, the plasma jet crosses the nozzle at lower applied powers. The minimum power or threshold power needed for the jet to cross the nozzle depends on the floating electrode width, i.e., the jet length improves with the floating electrode width for a fixed applied power. Figure 2 shows the variation of the jet length outside the nozzle with increasing applied power in the presence of floating electrodes of different widths. Without the additional floating electrode, the plasma jet crosses the nozzle at 34 W and reaches a maximum jet length of 22 mm at 75 W. The jet length saturates after 75 W. The threshold input power for the jet to cross the nozzle is 32, 31, 30, and 28 W for floating electrodes of widths 3, 5, 8, and 10 mm, respectively. Without a floating electrode, the jet has a length of 22 mm outside the nozzle at 75 W and this increased to 26 mm in the presence of a floating electrode of width 10 mm.

The net charge (Q) transferred from the electrode gap to the external circuit radially and the discharge power are measured in two methods. In the first method, the discharge current due to the oscillating motion of electrons between electrodes is calculated using Ohm’s law after scoping voltage across a 1 k$\Omega$ grounded resistor (r). The net charge is measured by time integrating this current signal. In the second method, a series 22 *pF* capacitor (C) is introduced at the ground line and records the voltage across the capacitor and, thereby, the net charge. The capacitor value is decided so that it can store all the charges that pass through the discharge during the one-half cycle of the applied voltage. The net charge Q measured through both methods are consistent with each other (Supplementary Fig. 1) and the estimated values from both methods suggest a 5–35 $\%$ decrement in the value of Q in the presence of additional floating electrodes of width 3–10 mm.

**Figure 3:**
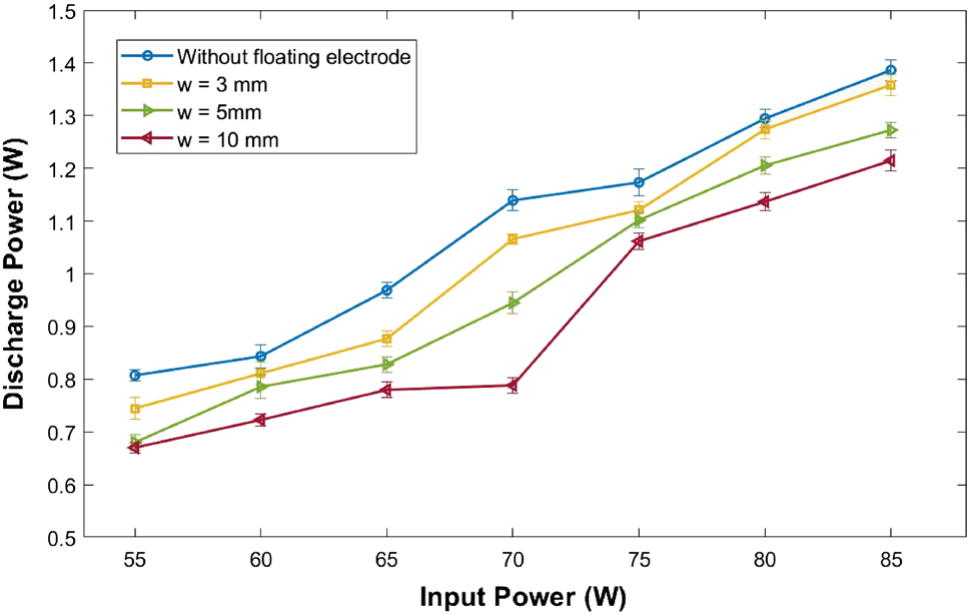
Plot of plasma discharge power versus input power for different floating electrode widths.

The discharge power or the electrical energy consumed by the discharge is estimated by calculating the area of the charge-voltage Lissajous curve, i.e., the plot of net charge measured across the capacitor series to the ground electrode versus applied voltage^53–55^. With increasing the floating electrode width, the shape of the charge-voltage Lissajous curve varies and the electrical energy consumed by the discharge decreases (Supplementary Fig. 2). Power consumed by plasma jets with additional floating rings of different widths as a function of input power is shown in Fig. 3. At a given input power, the plasma jet form a longer plume with less power consumption in the presence of a floating electrode.

Plasma jet current is measured by a current transformer placed around the plasma jet. To eliminate the chances of the formation of arcs and for the safe assembly of the current transformer, a glass tube of diameter 8 mm is placed at the plasma jet nozzle. This glass tube improved the jet length by 9 mm. The current transformer is then placed around the second glass tube, positioning the centre of the current transformer at 1.5 cm from the jet nozzle in the axial direction. The net charge transferred axially is estimated by time integrating the jet current signal. The net charge moving along the axial direction has increased by 16, 29, 43, and 59 $\%$ in the presence of additional floating electrodes of width 3, 5, 8, and 10 mm, respectively. The net charge transferred radially to the ground and the net charge flowing axially to the atmosphere are tabulated in Table 1.

**Table 1:** Charge dynamics in the presence of additional floating electrodes for different widths.

Floating electrode width (mm)	Charge transferred radially to ground ($10^{-9}$ C)	Charge transferred axially ($10^{-9}$ C)
0	2.233	0.802
3	2.118	0.933
5	2.05	1.038
8	1.747	1.150
10	1.642	1.283

**Figure 4:**
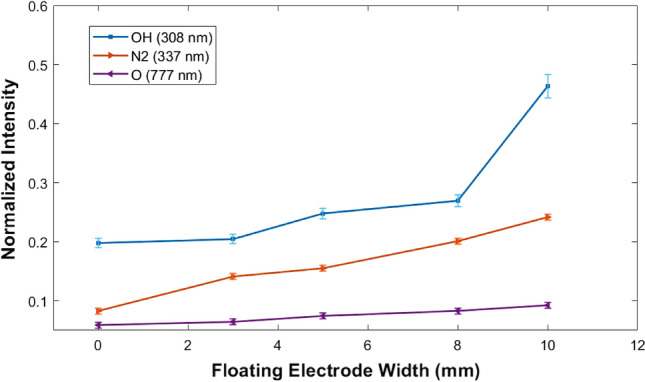
Variation in normalized optical emission intensity of reactive oxygen and nitrogen species in the presence of the floating electrode. (Emission intensity is normalized with respect to Ar (764 nm) line.

Qualitative analysis of species generation and the optical characteristics of the plasma is done by identifying different emission lines in the optical emission spectra obtained at different axial positions of the plasma plume. The time-integrated emission spectrum of the plasma plume is obtained at a position 15 mm axially and 4 mm radially (Supplementary Fig. 3). Optical emission spectra show that there is an increment in species emission intensity at the main discharge region as well as at the downstream region in the presence of the additional floating electrode. Figure 4 shows the variation in the relative intensity of *OH*, $N_{2}$, and *O* emission lines (needed for different kinds of applications) in the downstream region. Emission intensity is normalized with respect to Ar (764 nm) line.

**Figure 5:**
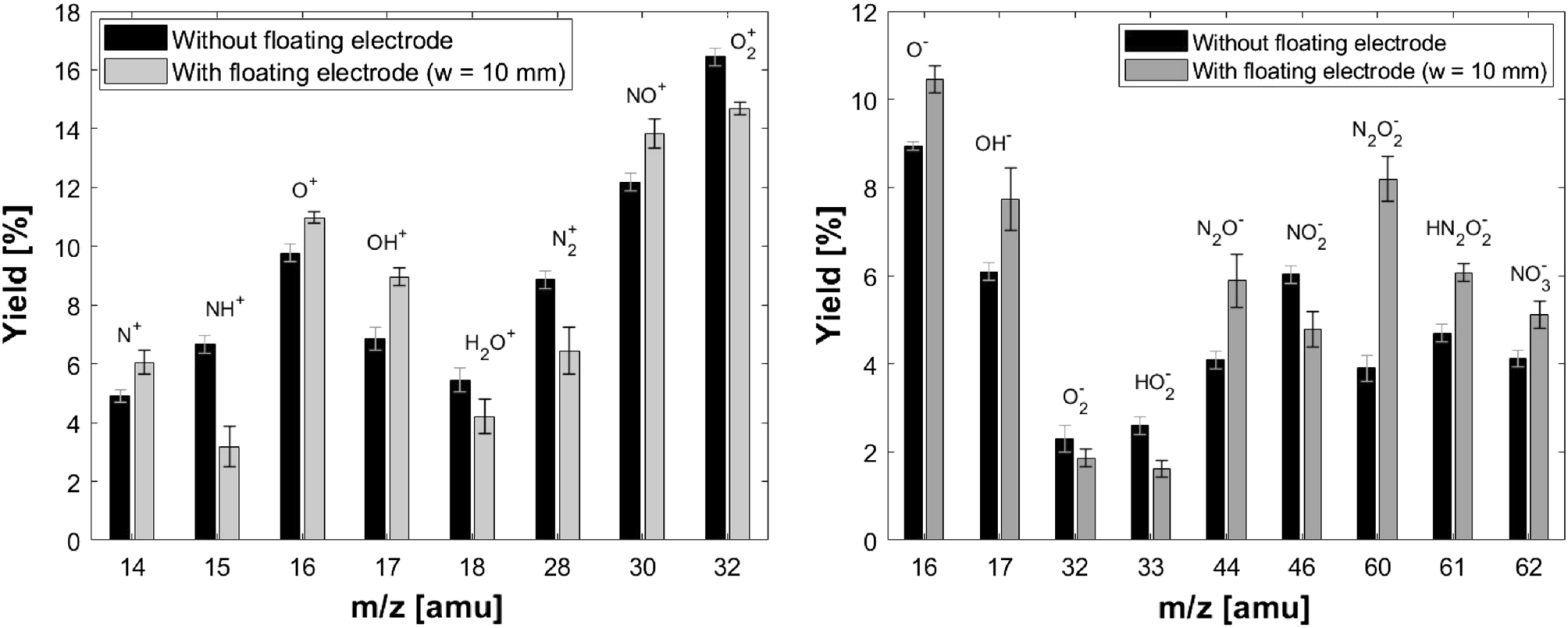
Relative yield of (**a**) positive ion and (**b**) negative ion in the absence of floating electrode and in the presence of additional floating electrode of width 10 mm.

The relative abundance of different positive and negative ion species in the plasma plume are analyzed from the mass spectra recorded with MBMS for a range of *m*/*z* = 1–100 (Supplementary Figs. 5, 6). It is observed that positive ions such as O$^{+}$ (9.7%), N$^{+}$ (4.98%), NH$^{+}$ (6.6%), OH$^{+}$ (6.8%), H$_{2}$O$^{+}$ (5.4%), N$_{2}^{+}$ (8.8%), NO$^{+}$ (12.1%), O$_{2}^{+}$ (16.4%), and negative ions such as O$^{-}$ (8.9%), OH$^{-}$ (7.7%), O$_{2}^{-}$ (2.3%), N$_{2}O^{-}$ (4%), NO$_{2}^{-}$ (6%), N$_{2}O_{2}^{-}$ (3.8%), HN$_{2}$O$_{2}^{-}$ (4.6%), NO$_{3}^{-}$ (4.1%) are the dominant species in the plasma plume. The species composition in the plasma plume depends on different atmospheric conditions, gas flow rate, plume–air interaction rate, applied power, and frequency. A quantitative analysis of positive and negative ion species in the plasma plume with MBMS at a constant operating condition shows that the relative yield of negative and positive ion species varies in the presence of floating electrodes of different widths, which is shown in Fig. 5. The targeted applications such as sterilization, surface modification, and material processing are highly sensitive to the active species composition in the plasma plume and demand a perfect tuning of the same. So any slight variation in the species composition in the plasma plume is effective in the application aspect. Analyzing the composition of ions in plasma help to investigate the possible plasma chemical models and to identify which dissociation channel may contribute to the production of desired reactive radicals. The relative yield of N$^{+}$, O$^{+}$, OH$^{+}$, NO$^{+}$, O$^{-}$, and OH$^{-}$, increased in the presence of the floating electrode. These ions are of interest for the treatment of biological samples. Reduction in the relative yield of N$_{2}^{+}$, H$_{2}$O$^{+}$, O$_{2}^{+}$, O$_{2}^{-}$, and NO$_{2}^{-}$ are complemented by the increment in the relative yield of N$^{+}$, OH$^{+}$, O$^{+}$, O$^{-}$ and OH$^{-}$, respectively. A detailed analysis of chemical kinetics is beyond the scope of this paper.

**Figure 6:**
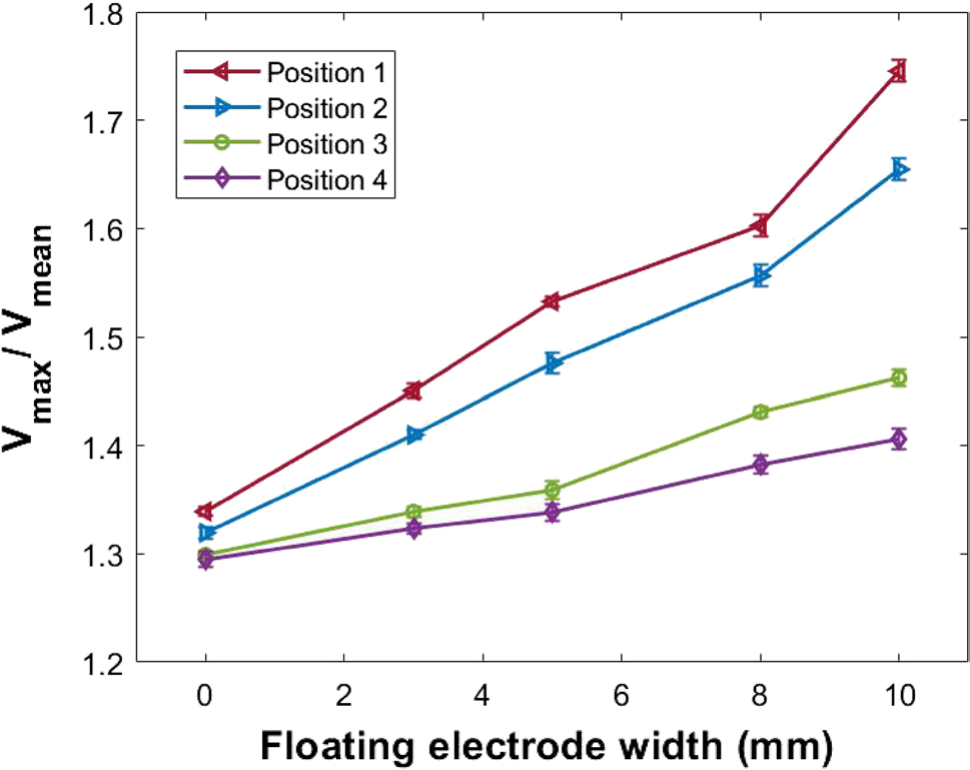
Variation in the ratio of maximum to mean of floating potential fluctuations with floating electrode width at different axial positions. Position 1 starts from the nozzle.

Electric field redistribution in the presence of the additional floating electrode needs to be investigated since the electric field impinging onto the target is also a crucial factor in practical application. The presence of a floating electrode cause changes in the charge distribution (Table 1) which can cause variations in the electric field. This changing electric field may cause fluctuations in the plasma potential. The observed increment in the gas temperature with floating electrode width could lead to thermal instability which will lead to fluctuations in floating potential^56^. These fluctuations in the potential and the non-uniformity of the electric field have an influence on the transport and heating of active species, the activation energy of the surface, and the adhesive ability of the treating surface. This may affect the biological or surface treatment by APPJ^49^. The possibility of potential fluctuations in the presence of the floating electrode in the jet propagation path is investigated with a single-pin probe at 4 axial positions (z = 2, 8, 14, and 20 mm). Floating potential measured with respect to the ground is analyzed with Fast Fourier Transform (FFT). The observed spectra are dominated by the power source frequency of 13.56 MHz. An infinite impulse response type notch filter has been designed and applied to eliminate the power supply frequency and its harmonics and to clearly observe other frequencies^57^ (Supplementary Fig. 7). The ratio of maximum to mean floating potential fluctuations, $V_{max}/V_{mean}$ (Fig. 6) at different axial positions are studied for jets with floating electrodes of different widths at constant input power (75 W) and gas flow rate (3 lpm). Prominent peaks of fluctuations are observed in the frequency range of 0.4–9 MHz and the amplitude of fluctuations increases with floating electrode width in the same frequency range. $V_{max}/V_{mean}$, which suggests the degree of fluctuation increased with the floating ring width and decreases along the plasma plume. The maximum degree of fluctuation is at the nozzle, close to the floating electrode. The frequency of fluctuation is independent of the floating electrode width.

**Figure 7:**
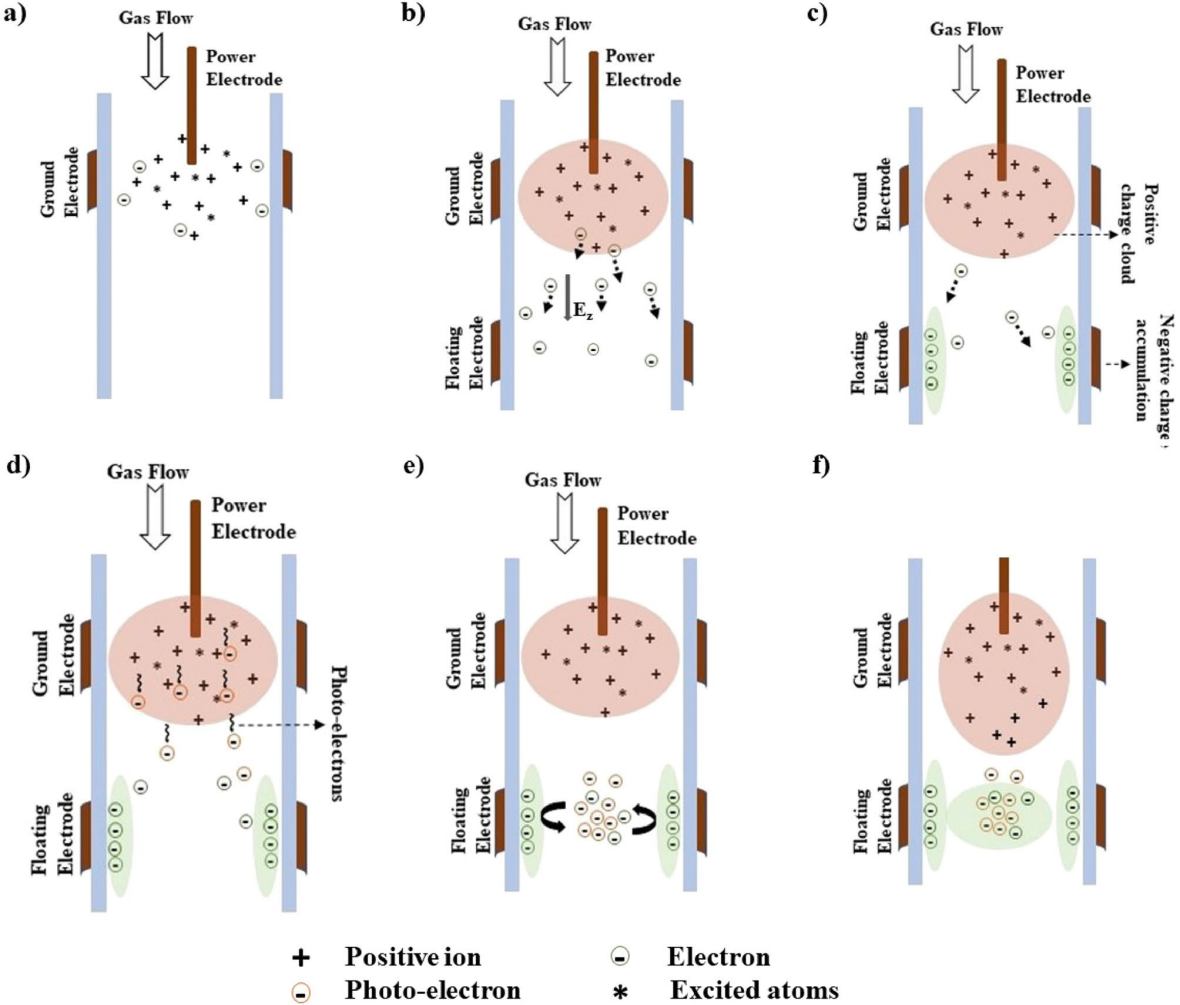
(**a**) Main discharge region with positive ions, excited atoms, and electrons that overcame the RF oscillations. (**b**) Electrons attract toward the floating electrode that is additionally introduced. (**c**) Electrons accumulate on the dielectric wall enclosing the floating electrode. (**d**) Photo-electrons generated in the positive charge cloud along with the primary electrons attract toward the floating electrode. (**e**) These electrons repel toward the center of the glass tube under the effect of the electric field generated by the electrons accumulated on the dielectric wall and get confined within the additional floating electrode. (**f**) These confined electrons form an electron cloud and act as an electron source ahead of ionization wave.

During this study, it is observed that the presence of an additional floating electrode in the path of APPJ with cross-field electrode configuration could improve the plasma jet length and decrease the threshold power needed for the jet to cross the nozzle. This is contrary to the reported results for an APPJ with linear field electrode configuration^43^, i.e., the jet needs high voltage to cross the floating ring inserted after the ground electrode. APPJs with cross-field electrode configuration have a high electric field in their electrode region, which suggests them as an effective jet design for gases with high breakdown voltages. But they have shorter and less reactive plasma plumes than linear field electrode configuration. Cross-field jets have an electric field in the radial direction. It is reported that the cross-field jet has a spatially confined gas ionization in the inter-electrode region due to the radially directed momentum imparted on the electrons by rapidly oscillating RF excitation voltage. Hence results in a shorter and less reactive plasma plume^26^. For cross-field configuration, generally, the RF field predominantly fills the inter-electrode region, and the fringe field extends to the lower region of electrodes in the absence of plasma. The RF electric field gets shielded out upon the ignition of the plasma and will be concentrated within the sheath between the power electrode and the surrounding bulk plasma. Therefore the RF electric field does not have a predominant effect in the region below inter-electrode space. The electrons and ions that overcome the rapid oscillations of the RF field in the inter-electrode space will move axially along with the feed gas flow (Fig. 7a). Since the ions carry the same electric charge as electrons and are much heavier, ions are much slower than electrons. By the conservation of energy and momentum, ions lose kinetic energy much more easily than electrons as they have similar mass as the gas molecule they collide with. Hence there will be a displacement of electrons with respect to ions. The displacement of electrons with respect to ions will generate a local electric field ($E_{z}$) and cause the drift motion of electrons in a direction opposite to this field. The additional floating electrode introduced after the ground electrode will initiate equal electron and ion currents. Being the lighter particle, the electrons will easily overcome the collisional strain compared to heavy ions on the way towards the floating electrode and form a layer of negative charge on the enclosing dielectric wall (Fig. 7b,c), i.e., electrons accumulate on the dielectric wall enclosed by the floating electrode leaving a positive space charge cloud behind with excited atoms and ions. Some of the excited atoms can emit short-wavelength ultraviolet radiation, and these photons create photo-electrons that will further attract toward the floating electrode^58^. Considering that the photo-electrons are emitted under the effect of short-wavelength ultraviolet radiation, they are expected to have high energy, and the minimum energy they can possess is comparable to that of thermal electrons. These high-energy photo-electrons could generate secondary electrons. The further coming thermal electrons along with photo-electrons and secondary electrons will then repel toward the center of the glass tube (where most of the driving gas is flowing) under the effect of the electric field due to the electrons accumulated on the floating electrode (Fig. 7d,e). A resultant force due to the flow towards the floating electrode and due to the electrostatic repulsion from the negative charge layer on the dielectric wall enclosing the floating electrode will impart on these electrons. A balance between this resultant force and the force caused by the drift velocity under the effect of $E_{z}$, is expected to confine the electrons near the floating electrode before they flew away and offer an enhanced electron population at the center of the glass tube which could intensify the ionization. The plasma jet can be described by an electrical circuit representing DBD discharge, considering the capacitance of the gas gap and the dielectric. The gas capacitance will be substituted by the resistance of plasma upon plasma ignition^59^. The ionization front in the capillary slows down upon reaching the nozzle because of the increase in capacitance with the change of capillary geometry^60^. The additional floating electrode confines the electrons before they flew away. This confined electron cloud near the nozzle will serve as an electron source ahead of the ionization wave and help to grow out of the nozzle with lower input powers (Fig. 7f). As this plasma does not satisfy the Meek breakdown condition ($\alpha d \ge 20$)^61^, the chance of streamer formation is very less. We considered the ionization length ($1 / \alpha$) to be 200 $\upmu$m^62^ and the inter-electrode spacing (*d*) is 3 mm which gives $\alpha d = 15$ and is less than 20. With increasing the floating electrode width, more electrons get confined at the centre of the glass tube where most of the driving gas is flowing. This will increase the plasma jet length (Fig. 2). The improved charge density at the centre of the glass tube will enhance the possibility of ionization and improve the plasma plume’s length and reactivity.

The decrement in the net charge (*Q*) transferred to the ground (radially) in the presence of the additional floating electrode (Table 1) suggests improved charge density in the discharge since the ions are considered quasi-immobile and the net charge (*Q*) is dominated by electron loss to the ground. These electrons and the photo-electrons created by the UV photons from the positive charge cloud in the main discharge get confined within the additional floating electrode and act as an electron source ahead of the ionization wave. A re-initiation of the ionization wave near the nozzle due to this confined electron cloud is suggested by the improved net charge in the plasma plume (Table 1) axially. This will enhance the chances of ionization as well as plasma jet length. The local field enhancement due to the electron cloud at the centre of the glass tube confined by the floating electrode results in a lower voltage needed for the plasma to sustain.

The jet length is almost the same at an input power of 50 W (Fig. 2). This suggests minimum electron confinement is needed to reinitiate the ionization wave at the nozzle for further propagation. Enhancement in the relative emission intensity of RONS from OES (Fig. 4) and the relative yield of positive and negative ions in the plume from MBMS (Fig. 5) justifies the improved reactivity of the plasma plume. The confined electron cloud at the center of the glass tube in the presence of the additional floating electrode helps to re-initiate the ionization wave at the nozzle and is suggested by the improved net charge in the plasma plume (Table 1). The improved electron density caused by the intensified ionization in the presence of an additional floating electrode and the improved plasma–air interaction rate due to the longer plume are expected to be the reason for the increase in the relative optical emission intensity of OH, N2, and O and for the variation in positive and negative ion species composition. The relative emission intensity of OH shows a drastic increment with floating electrode width and is expected to increase further up to a limit. We observed a slight increment in gas temperature with floating electrode width. Since gas temperature is another factor that plays a crucial role in the pathway of production of OH radicals, the increase in gas temperature also will affect the relative emission intensity of OH. For higher widths of the floating electrode, the combined effect is expected to be the reason for the increased relative intensity of OH (Fig. 4). After a limit, the OH density is expected to decrease due to thermal instability that can be caused by increased gas temperature^56^.

Potential fluctuations increase with the floating electrode width. The degree of fluctuation is maximum near the additional floating electrode and decreases along the plasma jet length. The floating potential fluctuations in the presence of the additional floating electrode could be related to the increased jet current due to intensified ionization and to the variation in the electric field caused by the redistribution of charge near the floating electrode. The thermal instability caused by an increment in gas temperature with floating electrode width also may initiate floating potential fluctuations. Fluctuations in the potential and the nonuniformity in the electric field can affect the plasma target interaction. Considering the desired sensitive applications, a further detailed study of the effect of floating electrode width on the potential fluctuations and optimization of floating electrode width with accurate gas temperature monitoring is needed.

## Conclusion

Atmospheric pressure plasma jets with cross-field electrode configuration has shorter and less reactive plasma plume. It is observed that the presence of an additional floating electrode can improve the ionization and can result in a longer and more reactive plasma plume. The decrement in the net charge transferred to the external circuit through the ground suggests improved electron density in discharge with less loss. These electrons along with the photo-electrons, get confined within the floating electrode and act as an electron source ahead of the ionization wavefront. This re-initiates the ionization wave that slows down at the nozzle and helps to increase the plasma jet length. Increment in the charge density, the normalized emission intensity of RONS, and the relative density of positive and negative ions in the plasma plume suggest improved plasma reactivity. The improved jet length which offers increased air interaction and increased ionization rate suggests this system as a better candidate for treating liquids and biological targets that cannot withstand a vacuum environment. Considering the increment in gas temperature and the fluctuations in floating potential and the necessity of better tuning of these factors for different applications, optimization of floating electrode width with accurate gas temperature monitoring is imperative.

## Supplementary Information

Supplementary Information.

## Data Availability

The datasets generated during and analyzed during the current study are available from the corresponding author upon reasonable request.

## References

[1] Tendero C, Tixier C, Tristant P, Desmaison J, Leprince P (2006). Atmospheric pressure plasmas: A review. Spectrochim. Acta Part B.

[2] Bárdos L, Baránková H (2010). Cold atmospheric plasma: Sources, processes, and applications. Thin Solid Films.

[3] Weltmann KD (2010). Atmospheric-pressure plasma sources: Prospective tools for plasma medicine. Pure Appl. Chem..

[4] Choudhury B (2023). Distributed compact plasma reactor decontamination for planetary protection in space missions. Sci. Rep..

[5] Xie J (2017). based plasma sanitizers. Proc. Natl. Acad. Sci..

[6] Kogelschatz U (2003). Dielectric-barrier discharges: Their history, discharge physics, and industrial applications. Plasma Chem. Plasma Process..

[7] Sekimoto S (2023). Flow control around NACA0015 airfoil using a dielectric barrier discharge plasma actuator over a wide range of the Reynolds number. Actuators.

[8] Roy S, Zhao P, DasGupta A, Soni J (2016). Dielectric barrier discharge actuator for vehicle drag reduction at highway speeds. AIP Adv..

[9] Winter J, Brandenburg R, Weltmann KD (2015). Atmospheric pressure plasma jets: An overview of devices and new directions. Plasma Sources Sci. Technol..

[10] Lu X, Laroussi M, Puech V (2012). On atmospheric-pressure non-equilibrium plasma jets and plasma bullets. Plasma Sources Sci. Technol..

[11] Das S, Gajula VP, Mohapatra S, Singh G, Kar S (2022). Role of cold atmospheric plasma in microbial inactivation and the factors affecting its efficacy. Health Sci. Rev..

[12] Yan D (2017). The specific vulnerabilities of cancer cells to the cold atmospheric plasma-stimulated solutions. Sci. Rep..

[13] Das S (2023). Antimicrobial efficacy of argon cold atmospheric pressure plasma jet on clinical isolates of multidrug-resistant ESKAPE bacteria. IEEE Trans. Radiat. Plasma Med. Sci..

[14] Barekzi N, Laroussi M (2012). Dose-dependent killing of leukemia cells by low-temperature plasma. J. Phys. D Appl. Phys..

[15] Lee HW, Nam SH, Mohamed AAH, Kim GC, Lee JK (2010). Atmospheric pressure plasma jet composed of three electrodes: Application to tooth bleaching. Plasma Processes Polym..

[16] Sangprasert, W., Nimmanpipug, P., Yavirach, P., Lee, V. S. & Boonyawan, D. Epoxy resin surface functionalization using atmospheric pressure plasma jet treatment. *Jpn. J. Appl. Phys.* **51**, 01AJ04 (2012).

[17] Kolacyak D, Ihde J, Merten C, Hartwig A, Lommatzsch U (2011). Fast functionalization of multi-walled carbon nanotubes by an atmospheric pressure plasma jet. J. Colloid Interface Sci..

[18] Nessim C, Boulos M, Kogelschatz U (2009). In-flight coating of nanoparticles in atmospheric-pressure DBD torch plasmas. Eur. Phys. J. Appl. Phys..

[19] Naseh MV (2010). Fast and clean functionalization of carbon nanotubes by dielectric barrier discharge plasma in air compared to acid treatment. Carbon.

[20] Mahreen PP, Kar S, Sahu J (2021). Atmospheric pressure non-thermal plasma in food processing. Food processing. Adv. Nonthermal Technol..

[21] Sohbatzadeh, F. *et al.* An innovative strategy to rapidly inactivate 8.2-log *Enterococcus faecalis* in fresh pineapple juice using cold atmospheric plasma. *Sci. Rep.* **11**, 16010 (2021). 10.1038/s41598-021-95452-2 PMC8346603 34362987

[22] Joshi D, Prakash GV, Ahammad SZ, Satyananda KAR, Sreekrishnan TR (2022). Development of low power non-thermal plasma jet and optimization of operational parameters for treating dyes and emerging contaminants. Plasma Sci. Technol.

[23] Park J, Henins I, Herrmann HW, Selwyn GS, Hicks RF (2001). Discharge phenomena of an atmospheric pressure radio-frequency capacitive plasma source. J. Appl. Phys..

[24] Abdel-Salam, M. In *High-Voltage Engineering: Theory and Practice, Revised and Expanded* 115–147 (CRC Press, 2018).

[25] Kunhardt EE (2000). Generation of large-volume, atmospheric-pressure, nonequilibrium plasmas. IEEE Trans. Plasma Sci..

[26] Walsh JL, Kong MG (2008). Contrasting characteristics of linear-field and cross-field atmospheric plasma jets. Appl. Phys. Lett..

[27] Lu X (2008). An 11 cm long atmospheric pressure cold plasma plume for applications of plasma medicine. Appl. Phys. Lett..

[28] Mahreen V, Prakash G, Kar S, Sahu D, Ganguli A (2021). Excitation of helical shape argon atmospheric pressure plasma jet using RF pulse modulation. J. Appl. Phys..

[29] Joh HM, Choi JY, Kim SJ, Chung TH, Kang TH (2014). Effect of additive oxygen gas on cellular response of lung cancer cells induced by atmospheric pressure helium plasma jet. Sci. Rep..

[30] Zhang M (2022). Generation of atmospheric pressure air diffuse discharge plasma in oxygen enriched working gas with floating electrode. Plasma Sci. Technol.

[31] Mahreen X, Prakash GV, Kar S, Sahu D, Ganguli A (2022). Influence of pulse modulation frequency on helium RF atmospheric pressure plasma jet characteristics. Contrib. Plasma Phys..

[32] Boselli M (2015). Characterization of a cold atmospheric pressure plasma jet device driven by nanosecond voltage pulses. IEEE Trans. Plasma Sci..

[33] Khan, M., Gajula, V. P., Kar, S., Sahu,D. P. & Ganguli, A. Modification of helical structures observed in pulsed RF argon plasma jets by alteration of modulation frequency and helium admixing. In *IEEE International Conference on Plasma Science (ICOPS), Seattle, WA, USA*. 10.1109/ICOPS45751.2022.9812966 (2002).

[34] Zhang S (2013). Comparison of atmospheric air plasmas excited by high-voltage nanosecond pulsed discharge and sinusoidal alternating current discharge. J. Appl. Phys..

[35] Nam SH (2013). High-efficiency tooth bleaching using non-thermal atmospheric pressure plasma with low concentration of hydrogen peroxide. J. Appl. Oral Sci..

[36] Liu CT (2014). Atomic oxygen and hydroxyl radical generation in round helium-based atmospheric-pressure plasma jets by various electrode arrangements and its application in sterilizing Streptococcus mutans. IEEE Trans. Plasma Sci..

[37] Yue Y, Pei X, Lu X (2016). OH density optimization in atmospheric-pressure plasma jet by using multiple ring electrodes. J. Appl. Phys..

[38] Mohamed AAH, Aljuhani MM, Almarashi JQ, Alhazime AA (2020). The effect of a second grounded electrode on the atmospheric pressure argon plasma jet. Plasma Res. Express.

[39] Ghimire B (2021). The influence of a second ground electrode on hydrogen peroxide production from an atmospheric pressure argon plasma jet and correlation to antibacterial efficacy and mammalian cell cytotoxicity. J. Phys. D Appl. Phys..

[40] Durscher, R. & Roy, S. On multi-barrier plasma actuators. In *49th AIAA Aerospace Sciences Meeting including the New Horizons Forum and Aerospace Exposition* 958 (2011).

[41] Zhang B, Ying Z, Feng L, Zhi FANG (2017). The influence of grounded electrode positions on the evolution and characteristics of an atmospheric pressure argon plasma jet. Plasma Sci. Technol.

[42] Liu CT (2016). Effect of ground and floating electrode on a helium-based plasma jet and its applications in sterilization and ceramic surface treatment. IEEE Trans. Plasma Sci..

[43] Zhu P (2017). Effect of external electric and magnetic field on propagation of atmospheric pressure plasma jet. Phys. Plasmas.

[44] Hu JT (2013). Effect of a floating electrode on a plasma jet. Phys. Plasmas.

[45] Bastami, A. & Ibrahim, A. Efficient radio frequency power generation and impedance matching (Doctoral dissertation, Massachusetts Institute of Technology) (2020).

[46] Xu W (2020). The activation of cancer cells by a nanosecond-pulsed magnetic field generator. J. Phys. D Appl. Phys..

[47] Lin L, Keidar M (2021). A map of control for cold atmospheric plasma jets: From physical mechanisms to optimizations. Appl. Phys. Rev..

[48] Kakei R, Ogino A, Iwata F, Nagatsu M (2010). Production of ultrafine atmospheric pressure plasma jet with nano-capillary. Thin Solid Films.

[49] Behmani D, Barman K, Bhattacharjee S (2021). Fluctuations of the plasma potential in atmospheric pressure micro-plasma jets. AIP Adv..

[50] Wenchao ZHU, Huang B, Ximing ZHU, Wencong CHEN, Yikang PU (2020). Investigation on the streamer propagation in atmospheric pressure helium plasma jet by the capacitive probe. Plasma Sci. Technol.

[51] Malović G, Puač N, Lazović S, Petrović Z (2010). Mass analysis of an atmospheric pressure plasma needle discharge. Plasma Sources Sci. Technol..

[52] Mahreen M, Ganguli A, Gajula VP, Kar S, Sahu D (2022). A joint calibration technique for improving measurement accuracy of voltage and current probes during synchronous operation for RF-based plasma devices. Rev. Sci. Instrum..

[53] Jovanović O, Nevena PUAČ, Škoro N (2022). A comparison of power measurement techniques and electrical characterization of an atmospheric pressure plasma jet. Plasma Sci. Technol.

[54] Wang WL (2016). Electrical and optical characteristics of the radio frequency surface dielectric barrier discharge plasma actuation. Chin. Phys. B.

[55] Hao, Z., *et al. * Optical and electric diagnostics of DBD plasma jet in atmospheric pressure Argon. In *IEEE International Conference on Condition Monitoring and Diagnosis*. (IEEE, 2012).

[56] Yue Y, Kondeti VSK, Sadeghi N, Bruggeman PJ (2022). Plasma dynamics, instabilities and OH generation in a pulsed atmospheric pressure plasma with liquid cathode: a diagnostic study. Plasma Sources Sci. Technol..

[57] Parks, T. W. & Burrus, C. S. *Digital Filter Design* (Wiley, 1987).

[58] Karakas E, Akman MA, Laroussi M (2012). The evolution of atmospheric-pressure low-temperature plasma jets: Jet current measurements. Plasma Sources Sci. Technol..

[59] Sobota A, Guaitella O, Rousseau A (2014). The influence of the geometry and electrical characteristics on the formation of the atmospheric pressure plasma jet. Plasma Sources Sci. Technol..

[60] Guaitella O, Sobota A (2015). The impingement of a kHz helium atmospheric pressure plasma jet on a dielectric surface. J. Phys. D Appl. Phys..

[61] Fridman, A. & Kennedy, L. A. *Plasma Physics and Engineering* 218–224 (CRC Press, 2021).

[62] Benilov MS (1999). Analysis of ionization non-equilibrium in the near-cathode region of atmospheric-pressure arcs. J. Phys. D Appl. Phys..

