# Supplementary material for: Effect of an additional floating electrode on radio frequency cross-field atmospheric pressure plasma jet
Source: Sci Rep. 2023 Jul 1;13:10665. doi: 10.1038/s41598-023-37805-7 (PMC10314906; doi:10.1038/s41598-023-37805-7)
Supplement: Supplementary file 1 — Supplementary Information. [file 41598_2023_37805_MOESM1_ESM.pdf]

Effect of an Additional Floating Electrode on Radio Frequency Cross-Field Atmospheric Pressure Plasma Jet

Radhika TP[1] and Satyanada Kar[1,*]

[1]Department of Energy Science and Engineering, Indian Institute of Technology Delhi, Hauz Khas, 110016, New Delhi, India
[*]satyananda@dese.iitd.ac.in

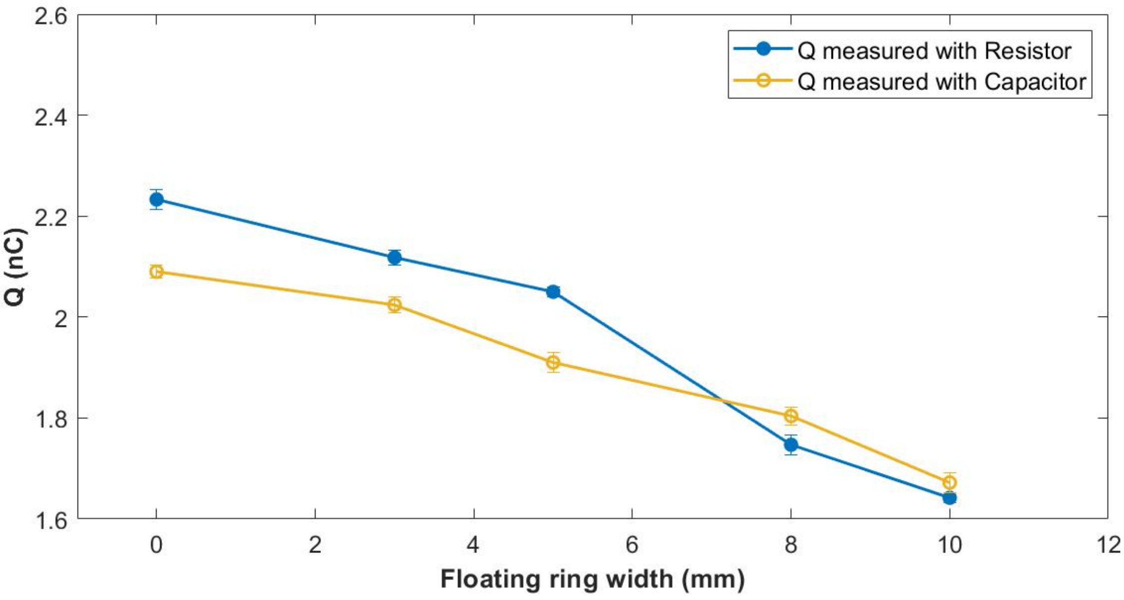

Supplementary Figure 1. Plot of net charge transferred from the electrode gap to the external circuit versus floating ring width estimated by the series resistor and series capacitor connected to ground at input power 75 W and gas flow of 3 lpm. The net charge Q estimated from both methods are consistent with each other and suggest a decrement of 5-35 % with a floating ring width of 3-10 mm

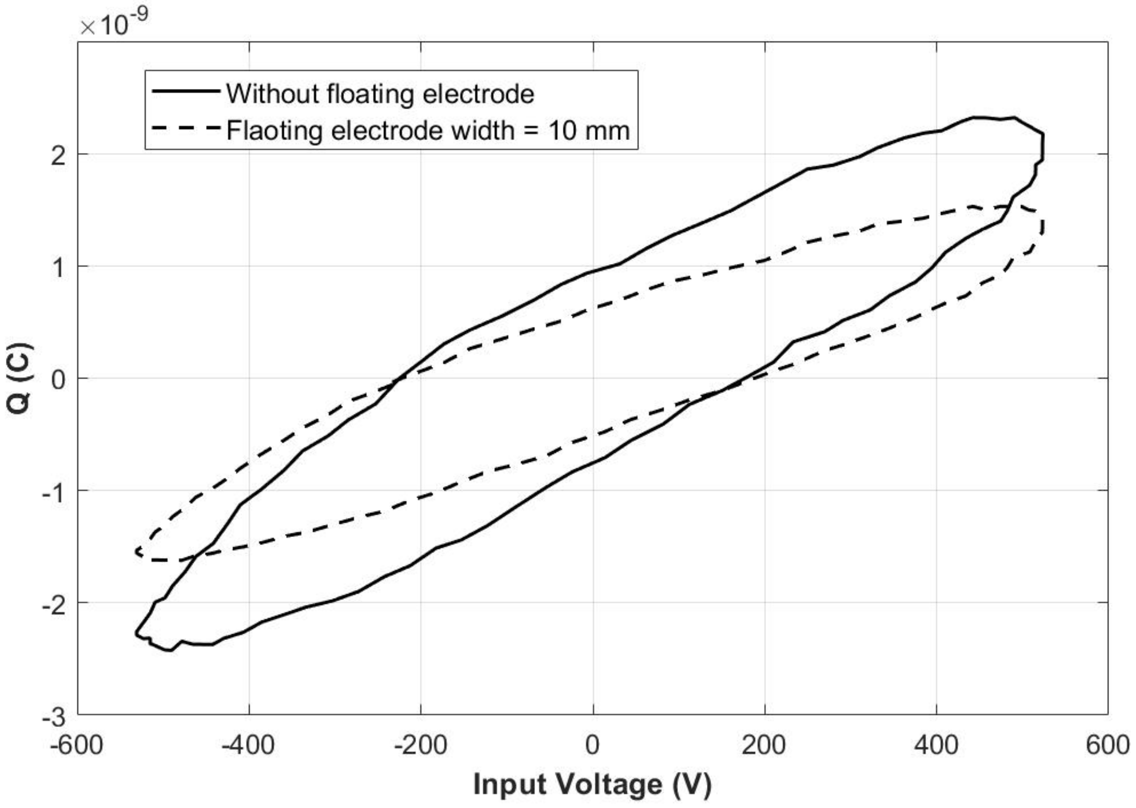

Supplementary Figure 2. Charge-Voltage Lissajous curves for plasma jets without additional floating electrode and with a floating electrode of width 10 *mm* at an input power of 70 *W*

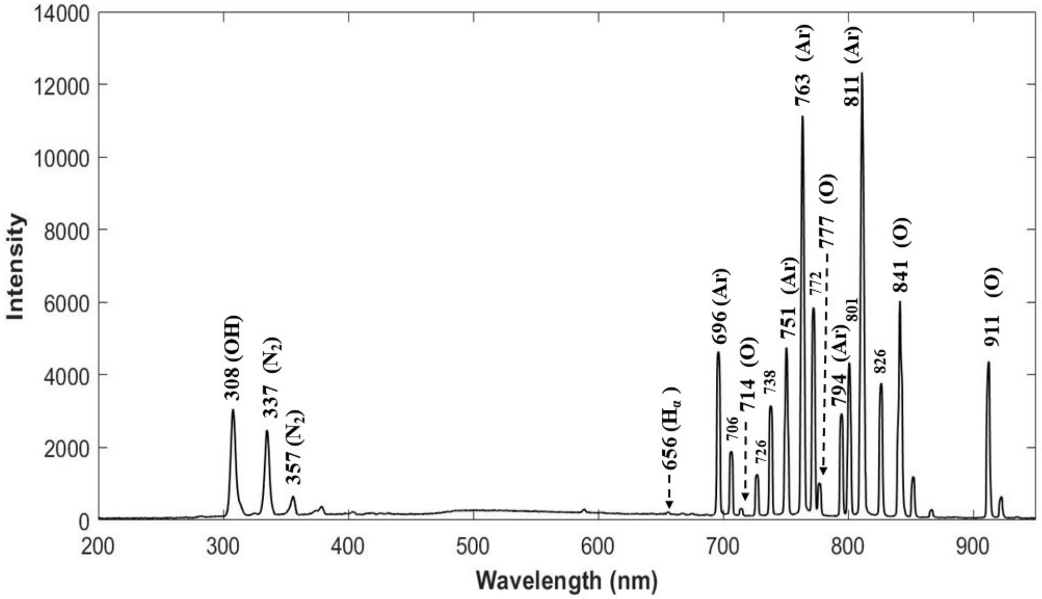

=3.25infigure1.eps

Supplementary Figure 3. Time integrated emission spectra of Argon plasma jet with input power of 75 W and gas flow of 3 lpm. The emission spectra of the plasma plume were obtained at a position 15 mm axially and 4 mm radially.

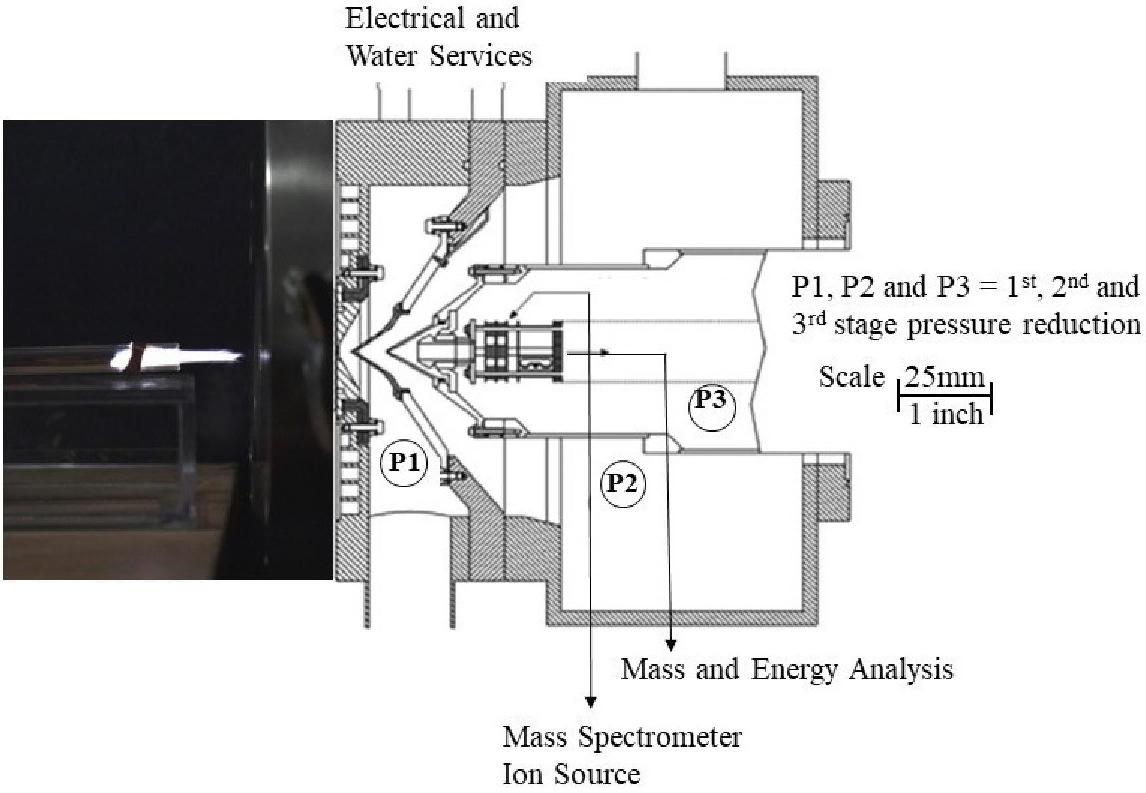

Supplementary Figure 4. Schematic representation of Atmospheric Pressure Plasma Jet assembly in front of the MBMS orifice for quantitative analysis of species composition in the plasma plume. MBMS schematic image taken from Malović, G. et al. The plasma jet was aligned perpendicular to the MBMS orifice at a fixed distance from the orifice and recorded the data in the range of 1-100 amu (m/z).

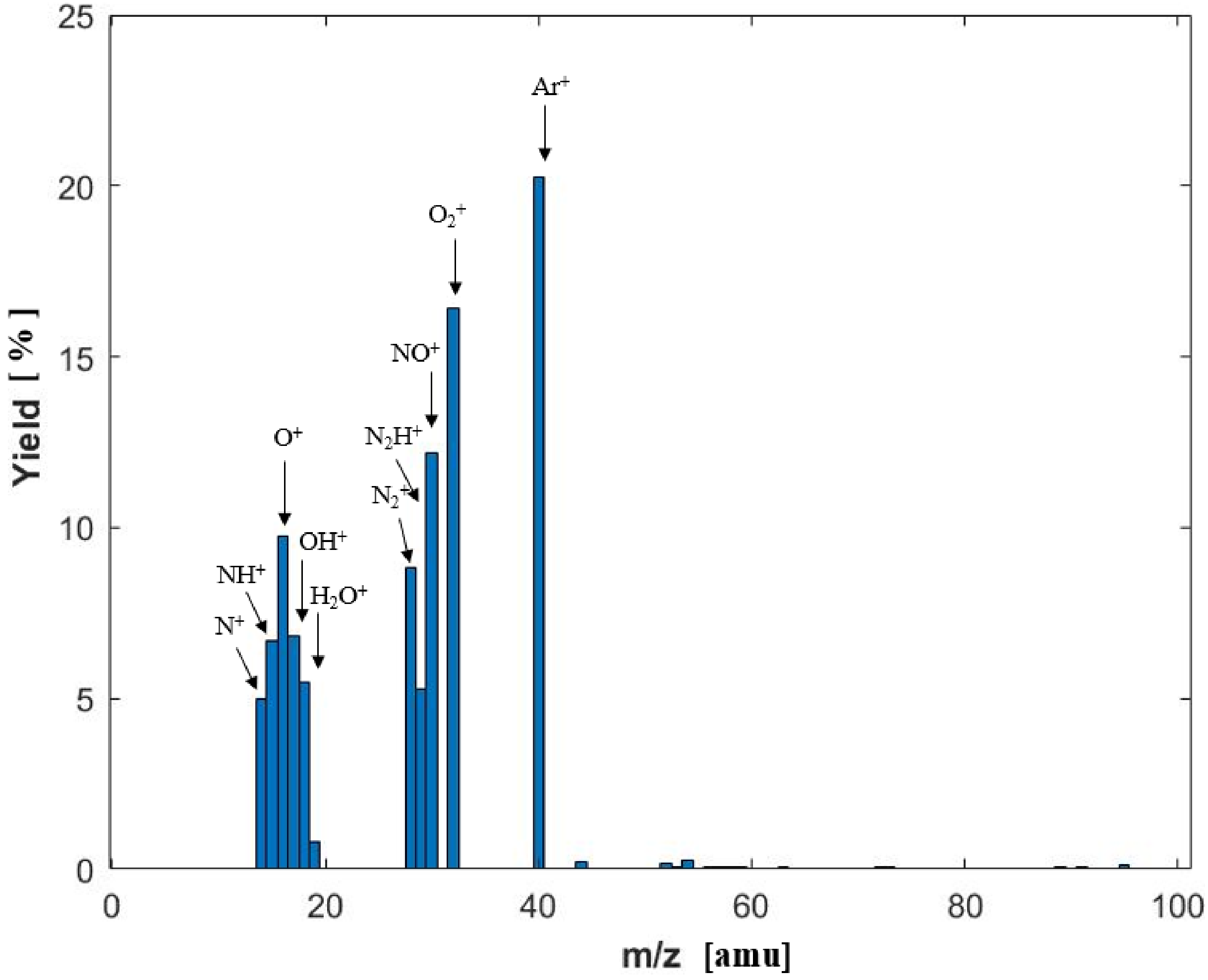

Supplementary Figure 5. Mass spectra of singly charged positive ion species obtained at an input power of 75 *W* and gas flow rate of 3 *lpm*

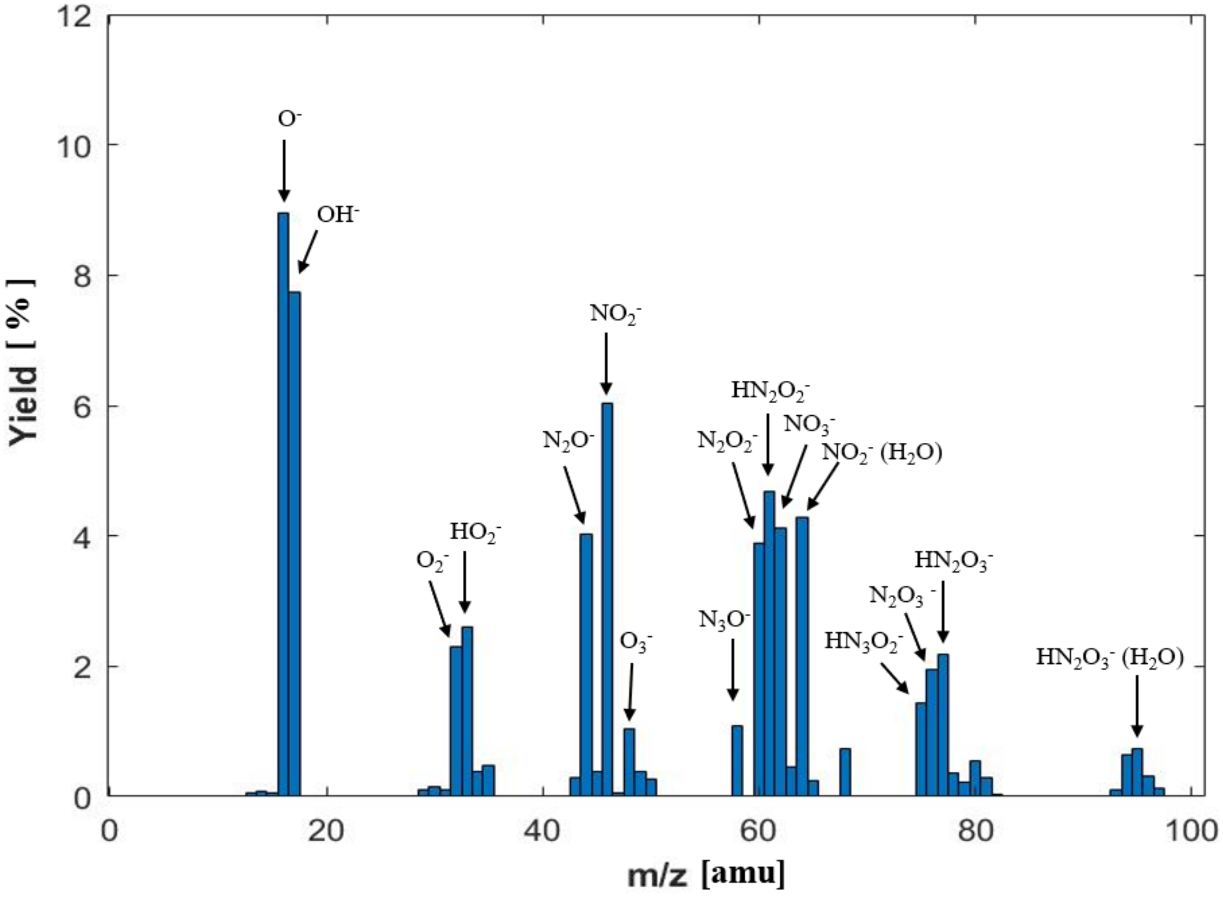

Supplementary Figure 6. Mass spectra of singly charged negative ion species obtained at an input power of 75 *W* and gas flow rate of 3 *lpm*

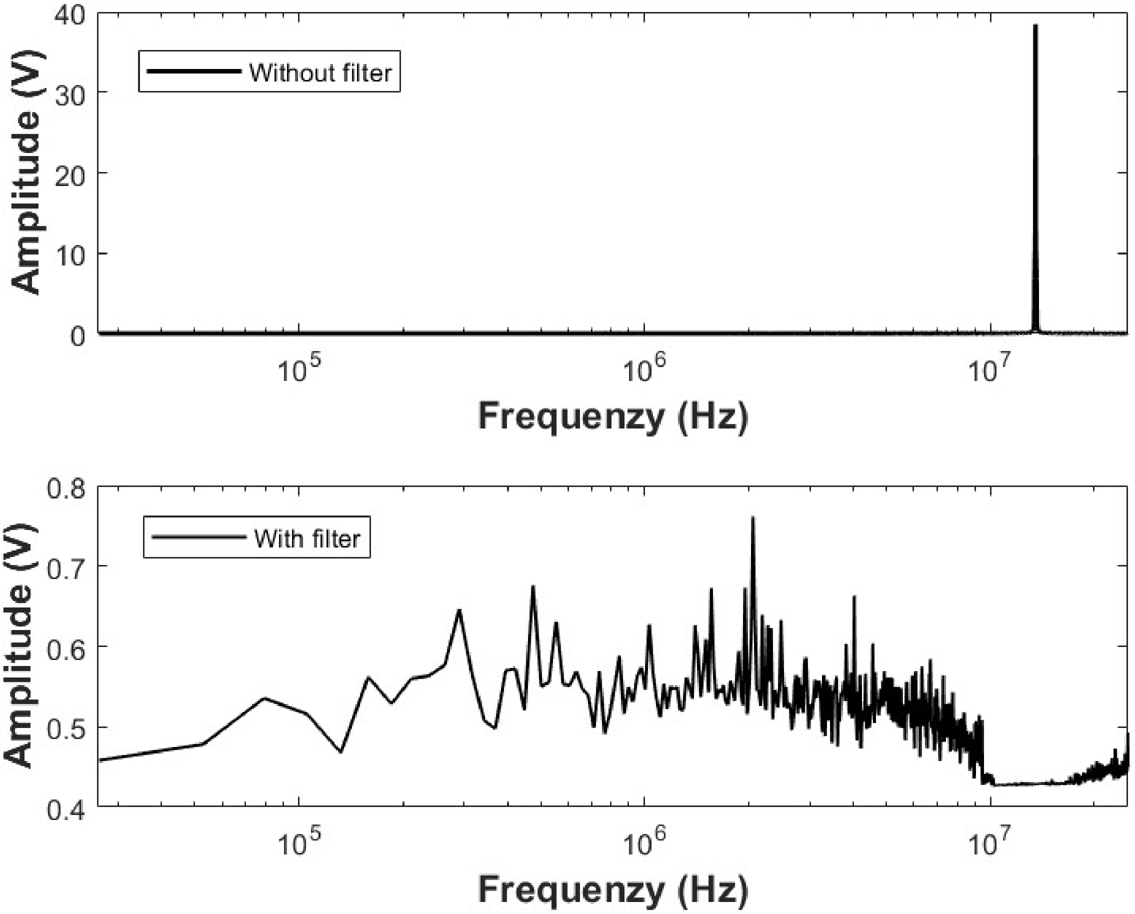

Supplementary Figure 7. FFT spectrum of the floating potential signal recorded at a position 2 *mm* in the axial direction and 4 *mm* in the radial direction from the jet nozzle in the presence of an additional floating electrode of width 10 *mm* a) without applying notch filter and b) with applying notch filter.
